# SARS-CoV-2 B.1.617.2 (Delta) Variant COVID-19 Outbreak Associated with a Gymnastics Facility — Oklahoma, April–May 2021

**DOI:** 10.15585/mmwr.mm7028e2

**Published:** 2021-07-16

**Authors:** Kendra Dougherty, Mike Mannell, Ozair Naqvi, Dakota Matson, Jolianne Stone

**Affiliations:** 1 Oklahoma State Department of Health.

The B.1.617.2 (Delta) variant of SARS-CoV-2, the virus that causes COVID-19, was identified in India in late 2020 and has subsequently been detected in approximately 60 countries (*1*). The B.1.617.2 variant has a potentially higher rate of transmission than other variants (*2*). During May 12–18, 2021, the Oklahoma State Department of Health (OSDH) Acute Disease Service (ADS) was notified by the OSDH Public Health Laboratory (PHL) of 21 SARS-CoV-2 B.1.617.2 specimens temporally and geographically clustered in central Oklahoma. Public health surveillance data indicated that these cases were associated with a local gymnastics facility (facility A). OSDH ADS and local health department staff members reinterviewed persons with B.1.617.2 variant–positive laboratory results and conducted contact tracing. Forty-seven COVID-19 cases (age range = 5–58 years), including 21 laboratory-confirmed B.1.617.2 variant and 26 epidemiologically linked cases, were associated with this outbreak during April 15–May 3, 2021. Cases occurred among 10 of 16 gymnast cohorts* and three staff members; secondary cases occurred in seven (33%) of 26 interviewed households with outbreak-associated cases. The overall facility and household attack rates were 20% and 53%, respectively. Forty (85%) persons with outbreak-associated COVID-19 had never received any COVID-19 vaccine doses (unvaccinated); three (6%) had received 1 dose of Moderna or Pfizer-BioNTech ≥14 days before a positive test result but had not received the second dose (partially vaccinated); four persons (9%) had received 2 doses of Moderna or Pfizer-BioNTech or a single dose of Janssen (Johnson & Johnson) vaccine ≥14 days before a positive test result (fully vaccinated). These findings suggest that the B.1.617.2 variant is highly transmissible in indoor sports settings and within households. Multicomponent prevention strategies including vaccination remain important to reduce the spread of SARS-CoV-2, including among persons participating in indoor sports^†^ and their contacts.

## Investigation and Findings

As of April 15, 2021, one Oklahoma resident with B.1.617.2 variant infection had been identified. During May 12–18, 2021, OSDH ADS received notification from OSDH PHL of 21 B.1.617.2 variant COVID-19 cases identified through virologic surveillance amplicon sequencing (*3*). Review of public health surveillance and investigation data revealed temporal and geographic clustering in central Oklahoma, and facility A was identified as a likely exposure site. OSDH ADS and local health department staff members reinterviewed persons with B.1.617.2 variant–positive laboratory results to verify exposure source, identified other settings with potential exposures, and conducted contact tracing. An outbreak-associated case was defined as 1) identification of the B.1.617.2 variant based on amplicon sequencing in a person with COVID-19 with an epidemiologic link to facility A, or 2) a COVID-19 case meeting the Council of State and Territorial Epidemiologists definition^§^ of a confirmed or probable case epidemiologically linked to a B.1.617.2 variant outbreak-associated case. Exposed contacts were defined as cohort members and staff members attending facility A during April 1–May 3 and household contacts of persons with outbreak-associated COVID-19.

To identify epidemiologically linked cases, contacts, and events where transmission might have occurred, OSDH ADS obtained a roster of gymnasts and staff members from facility A and a training and gymnastics meet schedule. The roster was compared against Oklahoma’s COVID-19 surveillance data to identify additional cases. The meet schedule was used to identify potential transmission settings, including other gymnastics facilities. Four Oklahoma patients with outbreak-associated COVID-19 attended two out-of-state regional gymnastics meets (April 15–18 and April 23–26) during their infectious period (Figure). OSDH ADS staff members used publicly available third-party websites listing gymnastics meet results to identify other potential exposures and cases within Oklahoma. The participant lists for Oklahoma gymnastics facilities obtained from the third-party websites were compared with Oklahoma’s COVID-19 surveillance data to ascertain whether secondary spread occurred at the two regional gymnastics meets. The COVID-19 immunization status of cases and contacts were verified using the state immunization registry. This activity was reviewed by CDC and was conducted consistent with applicable federal law and CDC policy.^¶^

**FIGURE:**
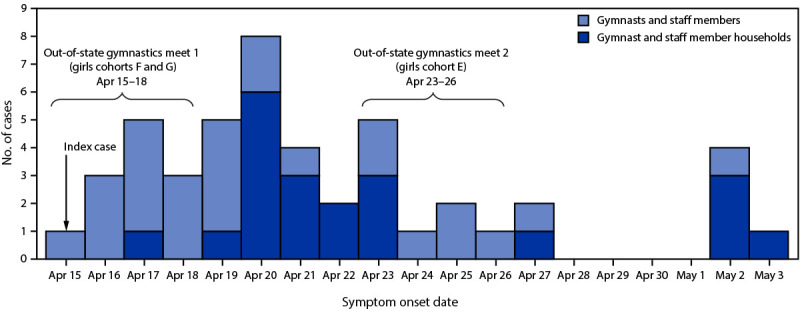
Symptom onset date* of COVID-19 cases associated with a SARS-CoV-2 B.1.617.2 (Delta) variant outbreak at gymnastics facility A (N = 47) — Oklahoma, April 15–May 3, 2021 * Or date of specimen collection, for asymptomatic or presymptomatic cases.

## Facility A Outbreak-Associated Cases

As of May 27, 2021, 47 COVID-19 cases associated with facility A were identified among 23 gymnasts, three staff members, and 21 of their household contacts. Among the 47 outbreak-associated cases, all 21 (45%) specimens available for sequencing were identified as B.1.617.2 variants. The facility A index case (gymnast) was identified on April 15, and cases occurred through May 3 (Figure). The median patient age was 14 years (range = 5–58 years) (Table 1). Approximately one half of cases occurred in females and two thirds in non-Hispanic White persons. Among all 47 cases, two adult patients were hospitalized (both unvaccinated and epidemiologically linked); one required intensive care. Review of meet schedules did not identify an exposure source outside facility A that explained the distribution of cases. Secondary spread was not identified among Oklahoma gymnasts participating at either regional gymnastics meet. No patient reported international travel in the 14 days preceding symptom onset (or date of specimen collection among presymptomatic and asymptomatic persons).

**TABLE 1:** Characteristics of gymnastics facility A–associated COVID-19 cases caused by SARS-CoV-2 variant B.1.617.2 (Delta) (N = 47) — Oklahoma, April–May 2021

Characteristic	No. (%)
**Case category**	
Gymnast	23 (49)
Staff member	3 (6)
Household contact	21 (45)
**Age group, yrs**	
Median (range)	14 (5–58)
<12	11 (23)
12–19	20 (43)
20–49	12 (26)
≥50	4 (9)
**Sex**	
Female	25 (53)
Male	22 (47)
**Race/Ethnicity**	
White, non-Hispanic	32 (68)
**Outcome**	
Total hospitalized	2 (4)
Intensive care unit	1 (2)
**Vaccination status**	
Unvaccinated*	40 (85)
Partially vaccinated^†^	3 (6)
Fully vaccinated^§^	4 (9)

* No COVID-19 vaccine doses received.

^†^ Receipt of 1 dose of Moderna or Pfizer-BioNTech vaccine ≥14 days before a positive SARS-CoV-2 test result, but not the second dose.

^§^ Receipt of 2 doses of Moderna or Pfizer-BioNTech vaccine or a single dose of Janssen vaccine ≥14 days before a positive SARS-CoV-2 test result.

## COVID-19 Vaccine Age-Eligibility and Vaccination Status

Among 194 identified exposed persons,** 74 (38%) were age-eligible to receive a COVID-19 vaccination by April 15, when the outbreak began. Among these vaccine-eligible persons, 17 (23%) were fully vaccinated, including four (9%) mildly symptomatic, of the 47 persons with outbreak-associated COVID-19; three had received Moderna vaccine and one Pfizer-BioNTech. Of the remaining 43 persons with outbreak-associated COVID-19, 40 (85%) were unvaccinated, and three (6%) were partially vaccinated; 27 (63%) were not age-eligible for vaccination when the outbreak began.

## B.1.617.2 Variant Transmission Summary

During April 15–May 3, 2021, COVID-19 cases occurred in 10 of 16 cohorts, including four of nine male cohorts and six of seven female cohorts, and three staff members. Among cohorts with identified cases, attack rates ranged from 8% to 60% (Table 2) (median = 32% overall; 42% [male]; 20% [female]). Among 26 identified households with cases, five (19%) were lost to follow-up, and no secondary cases were reported from 14 (54%). Among seven households with known secondary transmission, attack rates ranged from 80% to 100%. The overall facility-associated attack rate among 194 exposed persons was 24%, including 26 of 133 (20%) gymnasts and staff members and 42 of 80 (53%) household contacts.

**TABLE 2:** COVID-19 attack rates among gymnasts, staff members, and household contacts associated with a SARS-CoV-2 B.1.617.2 (Delta) variant outbreak at facility A — Oklahoma, April 15–May 3, 2021

Group	Total no. exposed*	No. of outbreak cases,^†^ attack rate (%)
**Overall**	**194**	**47 (24.2)**
**Gymnasts^§^ and staff members**	133	26 (19.5)
Girls	64	13 (20.3)
Girls cohort A	12	0 (—)
Girls cohort B	13	3 (23.1)
Girls cohort C	6	1 (16.7)
Girls cohort D	3	1 (33.3)
Girls cohort E	10	6 (60.0)
Girls cohort F	8	1 (12.5)
Girls cohort G	12	1 (8.3)
Boys	58	10 (17.2)
Boys cohort A	12	0 (—)
Boys cohort B	10	0 (—)
Boys cohort C	6	3 (50.0)
Boys cohort D	1	0 (—)
Boys cohort E	3	1 (33.3)
Boys cohort F	6	3 (50.0)
Boys cohort G	10	3 (30.0)
Boys cohort H	1	0 (—)
Boys cohort I	9	0 (—)
Staff members	11	3 (27.3)
**Household contacts^¶^**	80	42 (52.5)

* Exposed persons were defined as gymnast cohorts and staff members identified as attending facility A, along with any household contacts of outbreak cases.

^†^ An outbreak-associated case was defined as 1) identification of the B.1.617.2 variant based on amplicon sequencing in a person with COVID-19 with an epidemiologic link to facility A or 2) having epidemiologic linkage to a B.1.617.2 variant outbreak-associated case and classified as a confirmed or probable case per the Council of State and Territorial Epidemiologists COVID-19 Interim Case Definition, approved August 5, 2020. https://ndc.services.cdc.gov/case-definitions/coronavirus-disease-2019-2020-08-05/

^§^ A cohort is a group of gymnasts grouped by skill level and gender of which there were 16 in facility A. Each cohort had a designated practice schedule and had limited interaction with other cohorts within facility A.

^¶^ Household contacts include both outbreak-associated cases and exposed contacts from 21 of the 26 interviewed households. The remaining five households and outbreak-associated cases lost to follow-up were excluded from these numbers. Secondary cases were not identified in 14 of 21 interviewed households.

Several potential risk factors for transmission were identified through household interviews and direct observations at facility A, including nonadherence to recommended quarantine and testing guidance; delayed recognition of infection because of mild symptoms or attribution of symptoms to other causes; not using masks among active participants, coupled with increased respiration during active sport participation (further, facility A policy was that all persons not actively participating wear masks, but this policy was not always observed); poor facility ventilation; staff members training multiple cohorts; low COVID-19 vaccination coverage among participants (partly related to age eligibility); inadequate cleaning of high-touch surfaces between participant use; and overlapping cohort practice times, facilitating transmission between cohorts. The facility attempted to mitigate transmission by temporarily excluding cohorts with cases and requiring a negative test or 10-day quarantine before return. Other facility A mitigation measures were restricting locker room access, physical distancing in personal belonging storage area, and physical distancing when cohorts moved between practice locations.

## Public Health Response

On May 18, Epi-X, CDC’s Epidemic Information Exchange, was used to notify other states with gymnasts present at the two regional gymnastics meets of the potential exposure risk. A letter was distributed to facility A staff members and parents of gymnasts to notify them of the outbreak and to provide public health information regarding COVID-19. On-site testing and vaccination clinics offered to gymnasts, staff members, and household members at the facility resulted in one person being tested and nine vaccinated.

## Discussion

The primary case in this outbreak likely occurred in one or more staff members or gymnasts with undetected SARS-CoV-2 infection during April 1–13, 2021. Review of other potential exposure sources, including gymnastics meets, did not explain the case distribution among cohorts early in the outbreak because a limited number of cohorts participated in meets during this period.

Among the 47 outbreak-associated cases, sequencing of virus from the 21 patients with available specimens identified the B.1.617.2 (Delta) variant in all 21. Emerging evidence suggests that attack rates for the B.1.617.2 variant are potentially higher than are those for other variants of concern, including the B.1.1.7 (Alpha) variant (*2*,*4*). The 20% attack rate among exposed gymnasts and staff members in this outbreak was consistent with attack rates previously reported among persons in other high-risk activities (*5*–*7*), demonstrating that this variant is easily transmissible in high-risk settings with suboptimal adherence to recommended disease control measures. Further, household attack rates in this outbreak (53%) were higher than reported secondary attack rates associated with other SARS-CoV-2 lineages (17%)^††^ (*8*).

The findings in this report are subject to at least six limitations. First, interviews were only conducted with patients with reported positive SARS-CoV-2 test results associated with facility A. Other gymnasts and staff members who were exposed to patients from the facility and who might have had asymptomatic or mildly symptomatic infections were not interviewed or tested, which could have led to underascertainment of cases. Second, the number of cases might be underestimated if cases were not reported to the state surveillance system. Third, voluntary interviews and persons lost to follow-up or who refused to be interviewed could have resulted in underreporting of contacts, contact details, and patient details. Fourth, lags in reporting of state immunization registry data might have resulted in incomplete vaccination ascertainment. Fifth, amplicon sequencing was completed for fewer than one half of outbreak cases. Finally, vaccine effectiveness could not be calculated because of an inability to interview all persons associated with the outbreak and incomplete state immunization registry data.

These findings suggest that the B.1.617.2 variant is highly transmissible in indoor sports settings and households, which might lead to higher attack rates among exposed persons. Although the actual effectiveness of COVID-19 vaccines against the B.1.617.2 variant is not known at this time, current evidence indicates that vaccines approved under Emergency Use Authorization in the United States are effective against the variant. All eligible persons, including athletes and athletic staff members, should receive COVID-19 vaccination, especially those engaging in strenuous sports with limited ability to maintain physical distancing (*9*). In addition, multicomponent prevention strategies (e.g., testing, symptom monitoring, and other setting-specific measures) remain important to reduce SARS-CoV-2 transmission among persons participating in indoor sports and their contacts.

**Summary**

**What is already known about this topic?**

The SARS-CoV-2 B.1.617.2 (Delta) variant emerged in India and is currently widespread. Evidence suggests that it is potentially more transmissible than other variants.

**What is added by this report?**

During April 15–May 3, 2021, 47 COVID-19 cases were linked to a gymnastics facility, including 21 laboratory-confirmed B.1.617.2 cases and 26 epidemiologically linked cases. The overall facility and household attack rates were 20% and 53%, respectively.

**What are the implications for public health practice?**

The B.1.617.2 variant is highly transmissible in indoor sports settings and households, which might lead to increased attack rates. Multicomponent prevention strategies including vaccination remain important to reduce the spread of SARS-CoV-2 among persons participating in indoor sports and their contacts.

## Footnotes

* A cohort is a group of gymnasts grouped by skill level and gender of which there were 16 in facility A. Each cohort had a designated practice schedule and had limited interaction with other cohorts within facility A.

†
https://www.cdc.gov/coronavirus/2019-ncov/daily-life-coping/playing-sports.html

§ The Council of State and Territorial Epidemiologists COVID-19 Interim Case Definition, approved August 5, 2020, should be referenced for confirmatory and presumptive laboratory evidence definitions as well as confirmed and probable case classification definitions. https://ndc.services.cdc.gov/case-definitions/coronavirus-disease-2019-2020-08-05/

¶ 45 C.F.R. part 46, 21 C.F.R. part 56; 42 U.S.C. Sect. 241(d); 5 U.S.C. Sect. 552a; 44 U.S.C. Sect. 3501 et seq.

** Exposed persons were defined as gymnast cohorts and staff members identified as attending facility A along with any household contacts of outbreak cases.

††
http://medrxiv.org/lookup/doi/10.1101/2021.04.23.21255515
